# Antiviral activities of extremophilic actinomycetes extracts from Kazakhstan’s unique ecosystems against influenza viruses and paramyxoviruses

**DOI:** 10.1186/s12985-019-1254-1

**Published:** 2019-12-02

**Authors:** Vladimir Berezin, Diyora Abdukhakimova, Lyudmila Trenozhnikova, Andrey Bogoyavlenskiy, Aizhan Turmagambetova, Alpamys Issanov, Azliyati Azizan

**Affiliations:** 1 Research and Production Center for Microbiology and Virology, Almaty, Kazakhstan; 2 Nazarbayev University School of Medicine (NUSOM), Nur-Sultan, Kazakhstan

**Keywords:** Extremophiles, Actinomycetes, Antiviral inhibition, Hemagglutination activity, Neuraminidase activity, Antiviral drugs

## Abstract

**Background:**

Commercially available antiviral drugs, when used in the treatment of viral infections, do not always result in success. This is an urgent problem currently that needs to be addressed because several viruses including influenza and paramyxoviruses are acquiring multi-drug resistance. A potential solution for this emerging issue is to create new antiviral drugs from available compounds of natural products. It is known that the majority of drugs have been developed using compounds derived from actinomycetes, which are naturally occurring gram-positive bacteria. The purpose of this study was to investigate the antiviral properties of extremophilic actinomycetes extracts from strains that were isolated from extreme environments in Kazakhstan.

**Methods:**

Five strains of extremophilic actinomycetes isolated from the unique ecosystems of Kazakhstan were extracted and tested for antiviral activity against influenza viruses (strains H7N1, H5N3, H1N1 and H3N2) and paramyxoviruses (Sendai Virus and Newcastle Disease Virus). The antiviral activity of these selected extracts was tested by checking their effect on hemagglutination and neuraminidase activities of the studied viruses. Additionally, actinomycetes extracts were compared with commercially available antiviral drugs and some plant preparations that have been shown to exhibit antiviral properties.

**Results:**

The main findings show that extracts from strains K-192, K-340, K-362, K-522 and K525 showed antiviral activities when tested using influenza viruses, Sendai Virus, and Newcastle Disease Virus. These activities were comparable to those shown by Rimantadine and Tamiflu drugs, and “Virospan” and “Flavovir” plant preparations.

**Conclusions:**

We identified several extracts with antiviral activities against several strains of influenza viruses and paramyxoviruses. Our research findings can be applied towards characterization and development of new antiviral drugs from the active actinomycetes extracts.

## Background

Antibiotic-resistance is one of the most serious global health issues currently because the consequences are increased mortality and morbidity in humans and animals [1, 2]. However, the development of new antibiotics is compromised due to several complications such as challenges in finding natural compounds that possess characteristics suitable for a good pharmacological response when applied in the treatment of infections. There is also cost-effectiveness and cost-benefit issue that pharmacological companies face whereby the financial and other industrial resources outweigh the need for new drugs. Moreover, naturally derived compounds have complex chemical structures that make them difficult to first be discovered and then to create new drugs from these compounds [2]. The complexity of the studied preparations suggests the breadth of therapeutic use. Therefore, in our studies we investigated the presence of antiviral properties of new preparations that were isolated from extremophilic microorganisms.

Research highlights that drug-resistance in type A influenza virus is of high concern since it led to three pandemics in the twentieth century and can still do so in the future [3–5] . Genetic mechanisms that alter two main surface glycoproteins, hemagglutinin and neuraminidase caused the influenza viruses to develop resistance to already available antivirals [4]. Therefore, it was suggested that continuous antigenic variations in the virus can result in a new outbreak due to infections by the influenza virus [4].

Other viruses that serve as a threat to the animal populations come from the family *Paramyxoviridae*, specifically Newcastle disease virus and Sendai virus [6]. For example, the Newcastle disease virus is distributed worldwide and has the potential to cause large economic losses in the poultry industry [7, 8]. The Sendai virus, also known as murine and human parainfluenza virus type 1, severely infects the respiratory system of rodents and only mildly in humans, respectively [9]. Thus, it is crucial to undertake research that focuses on the development of new compounds for advancing drug-based therapies against viral infections such as influenza and paramyxoviruses.

Known sources for new antibiotics are naturally derived compounds; such compounds can be produced by actinomycetes. Actinomycetes are gram-positive bacteria that can be cultivated from soil and marine samples. Majority of actinomycetes can live in extreme environments, such as those found in saline soil distances and saltwater [10]. A vast majority of well-known anti-infective commercial drugs particularly antibiotics (such as streptomycin, neomycin and chloramphenicol) are derived from naturally produced compounds that originate from Actinobacteria particularly the actinomycetes strains from *Streptomyces* genus [11]. Several studies have reported actinomycetes producing novel metabolites with antiviral activities against several pathogenic viruses which include Western equine encephalitis virus, HIV-1, Zika virus, acyclovir-resistant herpes simplex virus type 1 as well as influenza A and B viruses [11–14].

The genomic RNA (3′-5) of Newcastle disease virus that is 15,186 nucleotides long encodes the nucleocapsid, P/V proteins, matrix or membrane protein, fusion protein, hemagglutinin-neuraminidase glycoprotein and large proteins [6, 15, 16]. The structure of genomic RNA of the Sendai virus, which is 15,384 nucleotides long, is like the Newcastle disease virus except in having the PCV proteins and hemagglutinin glycoprotein only [6]. From the structural composition of paramyxoviruses, we can see their similarity with influenza viruses in that their genomes also encode the hemagglutinin and neuraminidase glycoproteins. Therefore, the effects of actinomycetes extracts on these viruses in our study were checked by specifically assessing antiviral activity targeting these 2 glycoproteins. In this study, we investigated the antiviral properties of extremophilic actinomycetes extracts from strains that were isolated from extreme environments in Kazakhstan against the influenza viruses (strains H7N1, H5N3, H1N1, H3N2) and paramyxoviruses (Sendai Virus, Newcastle Disease Virus).

## Methods

### Cultivation and extraction of Actinomycetes

To study the antiviral properties of extremophilic actinomycetes extracts, 5 strains of extremophilic actinomycetes isolated from the unique ecosystems of Kazakhstan were selected: these are strains K-192, K-340, K-362, K-522, and K-525. Soil samples were collected from deserts, solonchaks, and forests in Almaty and Kostanay regions of Kazakhstan (Table 1).

**Table 1:**
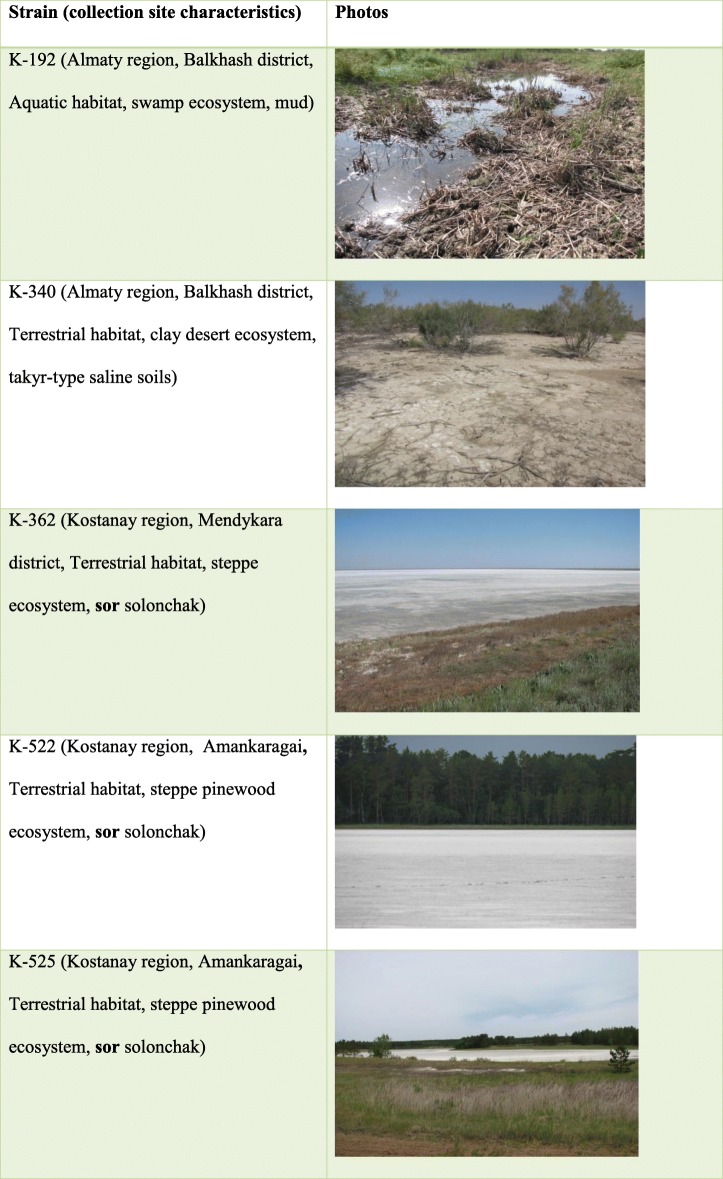
Characteristics of collection site of selected strains K-192 (a), K-340 (b), K-362 (c), K-522 (d), K-525 (e) from the extreme ecosystems of Kazakhstan. a. Almaty region, Balkhash district, Aquatic habitat, swamp ecosystem, mud; b. Almaty region, Balkhash district, Terrestrial habitat, clay desert ecosystem, takyr-type saline soils; c. Kostanay region, Mendykara district, Terrestrial habitat, steppe ecosystem, sor solonchak; d. Kostanay region, Amankaragai, Terrestrial habitat, steppe pinewood ecosystem, sor solonchak; e. Kostanay region, Amankaragai, Terrestrial habitat, steppe pinewood ecosystem, sor solonchak.

Five strains of actinomycetes, K-192, K-340, K-362, K-522, and K-525 were grown in liquid media in neutral (N) and extreme (S, for saline) conditions of cultivation. Two types of standard media were used to mimic neutral conditions of cultivation for strains K-340, K-362, and K-525 with the addition of components as listed below, per 1000 ml of culture media:

1. soy flour (12 g), glucose (12 g), and CaCO_3_ (2.5 g), adjusted to pH 7.2;
2. soluble starch (10 g), yeast extract (5 g), and casein hydrolyzate (10 g), adjusted to pH 7.2.

Neutral media for strain K-192 was composed of glucose **(**15 g), yeast extract (5 g), peptone (10 g) and the total media volume of 1000 ml was adjusted to pH 7.2. To mimic extreme conditions of cultivation, neutral media used for K-340, K-362, K-525 strains was additionally added NaCl (25 g), while the media for strain K-192 contained Na_2_CO_3_ (5 g and the media was adjusted to рН 9.0) per 1000 ml of culture media.

After 120 h of growth at 28 °C in a 100 ml total growth culture, the broth from 3 separate flasks were combined (3 flasks were cultured per strain) and separated from the mycelium (cells) by filtration through a coarse cloth using Büchner porcelain funnels in order to prepare cell-free culture broth. The rest of the actinomycetes cells were separated by centrifugation at 13,000 rpm. The native solution obtained after filtration and centrifugation was extracted using n-butanol (in a ratio 5:1). Extraction was further carried out in the refrigerator at 4 °C for 24 h. The n-butanol layer was separated on a separatory funnel and filtered through a filter paper. The collected n-butanol fraction was concentrated in a vacuum on a rotary evaporator “RV-10 Basic” to a dry residue, and the extracted substances were dissolved in chemically pure ethanol (80%) in an amount of 3 ml, and stored in the refrigerator until further analysis. Extracts prepared from culture broth are designated as “sample 1”; for example, culture broth from strain K-192 is labeled as “K-192-1”.

To prepare the extracts of actinomycetes cell biomass, the actinomycetes mycelium obtained after separation from the culture broth was relieved of moisture residues under pressure. The biomass wrung to 75% moisture were weighed and extracted with chemically pure ethanol (96%) in a ratio of 1:3, and the extracts were separated by filtration through a filter paper. The extraction was further carried out in the refrigerator for 24 h. The obtained extracts were concentrated in a vacuum on a rotary evaporator “RV-10 Basic” to a dry residue, and the extracted substances were dissolved in chemically pure ethanol (80%) in an amount of 3 ml and stored in the refrigerator. Extracts prepared from mycelium (or cell biomass) are designated as “sample 2”; for example, culture broth from strain K-192 is labeled as “K-192-2”.Thus, extracts of culture broth (CB), cell-free culture broth and cell biomass contained metabolites of extremophilic actinomycetes.

### Toxicity test of Actinomycetes

Primary toxicity analysis (hemolytic activity test) was first performed in virus-free environment, to investigate the possibility of actinomycetes extracts’ ability to kill tested cells using 3 models, specifically 2% chicken erythrocytes, chicken fibroblast cells, and 10-days chicken embryos.

First, hemolytic activity was tested on a 2% solution of chicken erythrocytes. The actinomycetes samples were mixed with chicken erythrocytes in a 1:5 ratio, and then incubated for 120 min at 37^0^С. Following incubation, an equal volume of cold buffered saline at pH 7.2 was added into the samples, which further underwent centrifugation at 13,000 rpm for 5 min. Supernatants were collected and the optical density of supernatant fluids was measured at 412 nm using spectrophotometer “Tecan M200” (Switzerland).

Second, toxicity analysis using chicken fibroblast cells (10^4^ cells/well) was estimated by the method of dehydrogenases activity detection (MTT-test), which is based on the ability of dehydrogenases of live cells to reduce the non-colored form of 3–4,5-dimetilazol-2,5-diphenyl tetrazolium (MTT-reagent) to the blue form of crystal formazan. MTT-reagent (Calbiochem, USA), which was dissolved in buffered saline was added in a concentration of 0.5 μg/ml. Then, the MTT solution (0.1 ml) was placed in the plate wells with cell culture preliminarily washed from the culture media. After 1-h incubation of MTT with cells, plate wells were washed with buffered saline and then 0.1 ml of dimethyl sulphoxide (DMSO) was added. Optical density in the wells was measured using the spectrophotometer “Tecan” M200 (Switzerland) at 535 nm.

Third, the toxicity of actinomycetes extracts was also investigated on the model of 10-days old chicken embryos following inoculation of 0.2 ml of tested preparations into the chorion-allantoic cavity of chicken embryos. Death of chicken embryos following 4 days incubation was used as a parameter for calculating the toxicity of the tested samples.

### Antiviral testing of Actinomycetes

Antiviral analysis was done to test the activity of the studied actinomycetes extracts on hemagglutination and neuraminidase activity of selected viruses. The action on the hemagglutination activity was tested using influenza viruses (strains H7N1, H5N3, H1N1, and H3N2) and paramyxoviruses (Sendai virus and Newcastle disease virus). The action of actinomycetes extracts on the neuraminidase activity on influenza viruses (strains H7N1, H5N3, H1N1, and H3N2) was also investigated. Further, the study of the virus-inhibition activity of 10 actinomycetes extracts on the influenza viruses (strains H7N1, H5N3, H1N1, and H3N2) and paramyxoviruses (Sendai virus and Newcastle disease virus) on the model of chicken embryos was performed. Finally, the virus-inhibition activity of the actinomycetes extracts was compared with preselected plant extracts (Virospan and Flavovir) and commercial antiviral drugs (Rimantadine and Tamiflu).

### Effect on hemagglutination activity

The following strains of viruses were grown in the allantoic cavity of 10-days old chicken embryos for 24–72 h at 37^0^С:

1. Orthomyxoviruses: avian influenza virus, strain A/tern/South Africa/1/61 (H5N3); human influenza virus, strain А/Almaty/8/98 (H3N2); avian influenza virus A/FPV/36/1 (H7N1), and human influenza virus, strain A/Vladivostok/2/09 (H1N1) (pandemic variant, resistant to Tamiflu);
2. Paramyxoviruses: avian paramyxovirus PMV-1/Beadette (Newcastle disease virus), and Sendai virus, strain 960.

Different doses of tested samples were mixed with equal volume of viruses with hemagglutination titer of 1:256. After 30 min incubation at 37 °C, hemagglutination activity was determined by a standard method using 0.75% erythrocyte solution [17, 18]. As a control, buffered saline, pH 7.2 was used. Hemagglutination inhibition activity was calculated by comparison of hemagglutination titers in a control and tested sample.

### Effect on neuraminidase activity

The same strains of orthomyxoviruses and paramyxoviruses were used in neuraminidase activity tests as in hemagglutination activity analysis. Also, the following reagents were used: enzyme buffer (32.5 mM MES, pH 6.0, Sigma, USA), substrate solution in enzyme buffer (0.2 mM MUNANA, 4 mM CaCl_2_, Sigma, USA), and stop-solution (25% ethanol, 0,1 M glycine, pH 10, Sigma, USA).

The reaction was carried out in the black, 96-well, flat-bottom plates with plastic lids (NUNC, USA). Tested viruses were diluted in a 1:5 ratio in enzyme buffer, followed by 7 four-fold dilutions in MES. Aliquots of 25 μl of each dilution were added to the wells. As a positive control, enzyme buffer were used, instead of virus-containing material.

After incubation for 30 min at 37 °C, 50 μl aliquot of substrate solution was added to each well and the plate was incubated at room temperature for 30 min. 150 μl aliquots of stop-solution buffer were added to the wells when incubation was complete, and optical density was measured by the “Tecan” spectrophotometer, using the fluorescent protocol [19, 20].

### Effect on virus-inhibitory activity

The specific virus-inhibitory activity of the studied preparations was determined in accordance with the guidelines of the “Pre-clinical Drug Research Guide” [21, 22]. Various doses of extract preparations obtained from extremophilic actinomycetes were mixed with an equal volume of 100 EID_50_/ml of the tested virus. After 30 min of incubation at 37^0^ С, the mixture was inoculated into the chorionic-allantoic cavity of 10-days old chicken embryos. Viruses were grown for 24–72 h at 37^0^С depending on the viral strain. The presence of the viruses was indicated by the hemagglutination test (HA-test). The ability of investigated preparations to inhibit virus infectious activity was estimated by comparison of the results of HA-test in experimental and control samples. As a control, the buffered saline pH 7.2, was used and the allantoic fluid with viruses but without the addition of preparations obtained from extremophilic actinomycetes.

### Comparison with selected antiviral drugs and plant preparations

In addition, the same procedure as in the testing of the effect of virus-inhibitory activity was applied using as a reference, the following preparations[23, 24]:

1. Rimantadine® (alpha-Methyl-1-adamantanemethylamine hydrochloride, “Olafarm”, Latvia);
2. Tamiflu® (3R,4R,5S)-4-acetylamino-5-amino-3(1-ethylpropoxy)-1-cyclohexene-1-carboxylic acid, ethyl ester, phosphate, Hoffmann-La Roche, Switzerland);
3. Selected earlier plant origin preparations “Virospan” and “Flavovir” of flavonoids and sesquiterpene nature with high antiviral activity.

### Data analysis

Before data analyses were performed and graphical representations prepared, the raw data were entered into the Excel software program after which the basic descriptive statistical analyses were performed. Based on optical density data, the mean values for the toxic dose of preparations that induced 50% lysis of erythrocytes, chicken fibroblasts and chicken embryos (ТC_50_) were calculated. The mean values (TC_50_) for the results of hemolytic activity testing were analyzed by Kruskal-Wallis rank test using the STATA 13 software.

The actions of extract preparations on the hemagglutination activity of test-viruses were calculated based on the average effective inhibition concentration of extract preparations (EIC_50_, mg/ml) capable of inhibiting 50% of viral hemagglutination activity (HI_50_). The same calculation procedure was applied to determine the capacity of tested preparations to inhibit 50% of neuraminidase activity (NI_50_).

As a criterion of specific antiviral action of tested preparations, the selective index (SI), determined by the ratio of average toxic substance concentration (TC_50_) to average effective virus-inhibiting concentration (EC_50_) was determined and used for comparison of antiviral activities.

## Results

### Cultivation and extraction of Actinomycetes strains

Thirty preparations were produced from 5 strains of actinomycetes numbered K-192, K-340, K-362, K-522, and K-525. These preparations were previously screened and shown to exhibit inhibitory bioactivities against other microbial pathogens [25]. The 30 preparations were labeled as listed below:

1. K-192-CBN, K-340-CBN, K-362-CBN, K-522-CBN, and K-525-CBN (“CBN” – Culture Broth Neutral): these are un-extracted samples of actinomycetes strain culture broth of strains grown in neutral condition of growth;
2. K-192-CBS, K-340-CBS, K-362-CBS, K-522-CBS, and K-525-CBS
  (“CBS” – Culture Broth Saline): these are un-extracted samples of culture broth of actinomycetes strains grown in the saline (extreme) condition of growth;
3. K-192-1 N, K-340-1 N, K-362-1 N, K-522-1 N, and K-525-1 N: these samples are extracts prepared from actinomycetes strain culture broth in neutral conditions of growth;
4. K-192-1S, K-340-1S, K-362-1S, K-522-1S and K-525-1S – these samples are extracts prepared from actinomycetes strain culture broth in saline (extreme) conditions of growth;
5. K-192-2 N, K-340-2 N, K-362-2 N, K-522-2 N, and K-525-2 N – these samples are extracts prepared from actinomycetes strain biomass (cells) in neutral conditions of growth;
6. K-192-2S, K-340-2S, K-362-2S, K-522-2S, and K-525-2S – these samples are extracts prepared from actinomycetes strain biomass (cells) in saline conditions of growth.

### Toxicity testing of Actinomycetes preparations

Toxicity of the 30 preparations described above were tested using three different assays; effect of the preparations to induce 50% lysis of erythrocytes (ТC_50_ mean value, mg/ml), effect of the preparations to induce 50% death of chicken fibroblast cells (ТC_50_ mean value, mg/ml) and effect of the preparations to induce 50% death of chicken embryos (ТC_50_ mean value, mg/ml). The results of investigation are presented in Additional file 1: Figure S1, Additional file 2: Figure S2 and Additional file 3: Figure S3. In these assays, the lower concentrations of preparations to induce the measured effect indicated a higher level of toxicity. We observed that the level of measured toxicity varied according to the doses of the preparations as well as the assays. Furthermore, the toxicity effects vary according to the different biological models used (when tested using either 2% chicken erythrocytes, chicken fibroblast cells, or 10-days chicken embryos) for the toxicity tests. Overall, lower toxicity was observed when preparations were tested on the chicken embryos, while higher toxicity in preparations tested on chicken fibroblast cells. Strain K-525 showed the least amount of sample preparation needed to kill 50% of the erythrocytes under neutral conditions, while CBS preparations required the highest concentration (TC_50_) to destroy 50% of the erythrocytes. Overall, the CBS and CBN as growth conditions resulted in lower toxicity of preparations since these samples required the highest concentrations to kill 50% of chicken fibroblasts. We found that actinomycetes grown in 2 N, 1 N, and 1S, and 2S conditions showed higher toxicity towards chicken fibroblasts when compared to culture broth preparations. The ability of sample preparations to induce the death of chicken embryos when tested showed that the 1 N preparation condition also showed high toxicity for preparations from all strains except for K-362. As expected, the preparations CBS showed relatively low toxicity in comparison to other conditions for all selected preparations. Furthermore, strain K-340 showed the lowest toxicity (highest TC_50_) for preparation 1S, when compared to preparations 1S for other strains which exhibited comparatively high toxicity. From the output of Kruskal-Wallis H test that was run for each individual strain separately used in hemolytic activity testing, it was shown that there was no statistically significant difference in toxicity between 6 different extracts, since all *p*-values were more than 0.05 (*p-*value = 0.4159) for all strains analyzed. The results of analysis are presented in Additional file 4: Table S1. The toxic dose of preparations that induced 50% lysis of erythrocytes, chicken fibroblasts and chicken embryos (ТC_50_) was determined and used to calculate the selective index (SI) that is represented in Table 2 (SI was determined by the ratio of average toxic substance concentration (TC_50_) to average effective virus-inhibiting concentration (EC_50_) ).

**Table 2:** Specific antiviral action (SI-index) of preparations obtained from extremophilic actinomycetes against ortho- and paramyxoviruses. SI was determined by the ratio of average toxic substance concentration (TC_50_) to average effective virus-inhibiting concentration (EC_50_). SI-index greater than or equal to 10 marked good antiviral action of actinomycetes extracts that specifically killed the virus. The actinomycetes extracts K-340-2S, K-362-2 N, K-362-1S, K-362-2S demonstrated high virus-inhibition activity against all investigated strains of influenza virus. Only the extracts K-192-1S and K-192-2S showed good SI against PMV-1/Beadette virus strain. For strain K-525 extract of biomass grown in saline conditions, K-525-2S, had higher SI indexes against tested viral strains than extracts from culture liquid or biomass extract grown in neutral condition. Overall, biomass extracts grown in saline conditions had better specific antiviral action than extracts grown from culture liquid in neutral conditions

Viruses	Influenza virus H3N2	Influenza virus H1N1	Influenza virus H5N3	Influenza virus H7N1	PMV-1/Beadette	Sendai. strain 960
Preparations	SI	SI	SI	SI	SI	SI
K-192-1S	10	10	2	10.5	10	11.76
K-192-2S	28.6	40	40	5	10	9.3
K-340-1 N	2	2	2	2	2	11.1
K-340-2S	28.6	28.57	13.3	40	2	1
K-362-2 N	12.5	40	10	40	2	5.4
K-362-1S	40	40	40	28.57	8.7	8.7
K-362-2S	40	40	40	40	2	11.76
K-522-CBN	2	2	2	2	2	13.3
K-522-1S	2	2	2	2	2	10
K-522-2S	10	5.88	2	11.76	2	2
K-525-CBN	2	2	2	2	2	10
K-525-1 N	2	2	2	10	2	2
K-525-2 N	2	25	2	2	2	11.1
K-525-1S	2	2	2	2	2	10
K-525-2S	2	40	28.57	33.3	2	14.29

### Effect of actinomycetes preparations on hemagglutination activity of selected influenza and paramyxoviruses

The influence of tested samples which are preparations of extremophilic actinomycetes (actinomycetes biomass extracts, culture broth extracts, and cell-free culture media) on the hemagglutination activity of orthomyxoviruses (influenza viruses strains H7N1, H5N3, H1N1, H3N2) and paramyxoviruses (NDV, Sendai virus) was studied. The effects of preparations on the hemagglutination activity of test-viruses were calculated to determine effective inhibition concentration of preparation (EIC_50_, mg/ml) that inhibited 50% of viral hemagglutination activity.

**Fig. 1:**
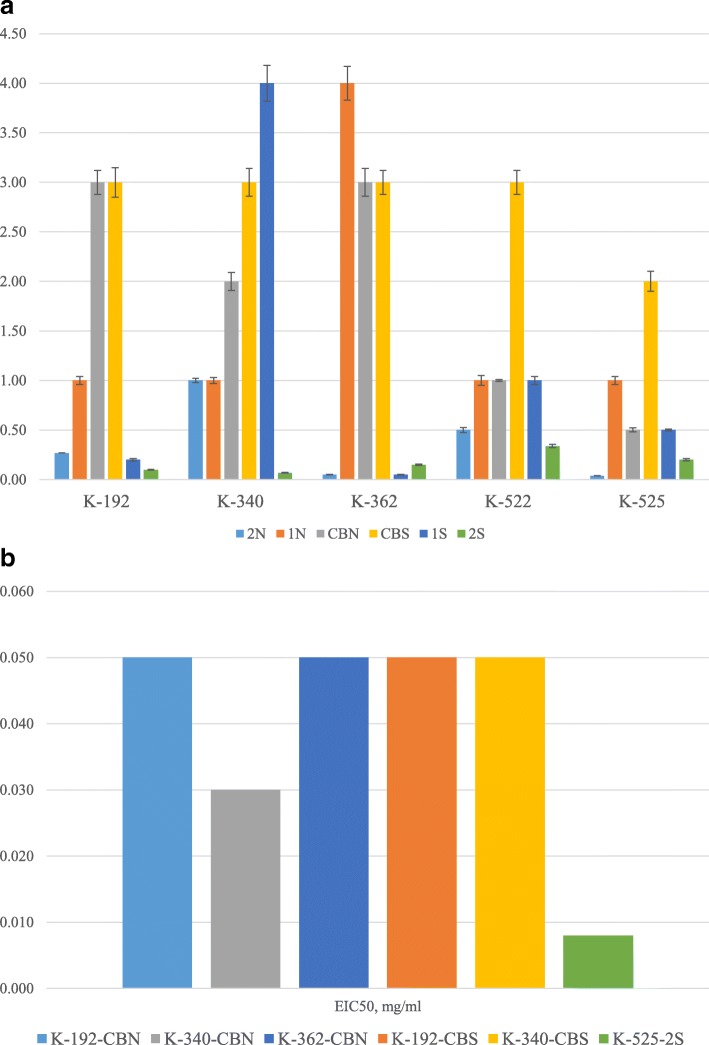
**a** The effect of preparations on the hemagglutination inhibition (HI mean, mg/ml) of influenza virus H1N1. Tested samples were mixed with equal volume of viruses with hemagglutination titer of 1:256. After 30 min incubation at 37 °C, hemagglutination activity was determined by a standard method [2, 4]. Hemagglutination inhibition activity was calculated by comparison of hemagglutination titers in the control and tested sample. **b** Effective Inhibition Concentration (EIC50, mg/ml) of six preparations against influenza virus H1N1

We determined that most of the extremophiles actinomycetes preparations did not inhibit hemagglutination activity of most of the tested influenza viruses (strains H7N1, H3N2, and H5N3) and paramyxoviruses. However, as shown in Fig. 1a and Fig. 1, several preparations were found to inhibit hemagglutination activity of influenza virus H1N1.

The quantitative measurement of specific inhibition of hemagglutination activity in influenza virus H1N1 by preparations from 5 selected strains of actinomycetes is indicated by mean and standard deviation measures of hemagglutination inhibition (HI) of the H1N1 virus (Fig. 1a). Thus, the lower the value for HI indicates better effectiveness of the preparation to inhibit the agglutinating activity of the studied virus. Preparations CBS of most strains required the highest concentrations for HI_50_ (hence less effective), while preparations 2S from strains K-192, K-340 and K-362, and preparation 2 N from strains K-362 and K-525 required the least concentration when compared with the other preparations (and thus more effective) to induce inhibition of the hemagglutination activity of the H1N1. Culture broth preparations CBN and CBS from strains K-192 and K-362 are similar in the ability to induce HI while CBN from K-340, K-522 and K-525 showed more effective inhibitory effects on hemagglutination activity. Comparing extracts prepared from culture broths of strains grown in a neutral condition (1 N) when compared to strains grown in saline condition (1S), preparations 1S are more effective than 1 N for strains K-192, K-362 and K-525. For preparations of biomass from strains grown in a neutral condition (2 N) and saline condition (2S), preparations 2S are more effective 2 N for strains K-192, K-340, and K-522.

Figure 1b summarizes 6 selected preparations from strains K-192, K-340, K-362 and K-525 grown in neutral or saline conditions that were effective in inhibiting 50% of the hemagglutination activity of the influenza virus H1N1. K-525-2S exhibited the lowest effective inhibition concentration and hence is the most effective preparation specifically at 0.008 mg/ml when compared to the other 5 selected preparations that inhibited 50% of hemagglutination activity of influenza virus H1N1.

### Effect of actinomycetes preparations on neuraminidase activity of selected influenza and paramyxoviruses

The effect of the tested preparations (extracts of extremophilic actinomycetes, cell-free culture fluid) on neuraminidase activity of influenza A viruses with various types of neuraminidases (N1, N2, N3), including strains belonging to epidemically significant strains and strains resistant to commercial antiviral drugs, was studied. The capacity of tested preparations to inhibit of neuraminidase activity of influenza viruses was estimated as effective concentration of tested preparations (EIC_50_, mg/ml) capable to inhibit 50% of neuraminidase activity (NI_50_).

**Fig. 2:**
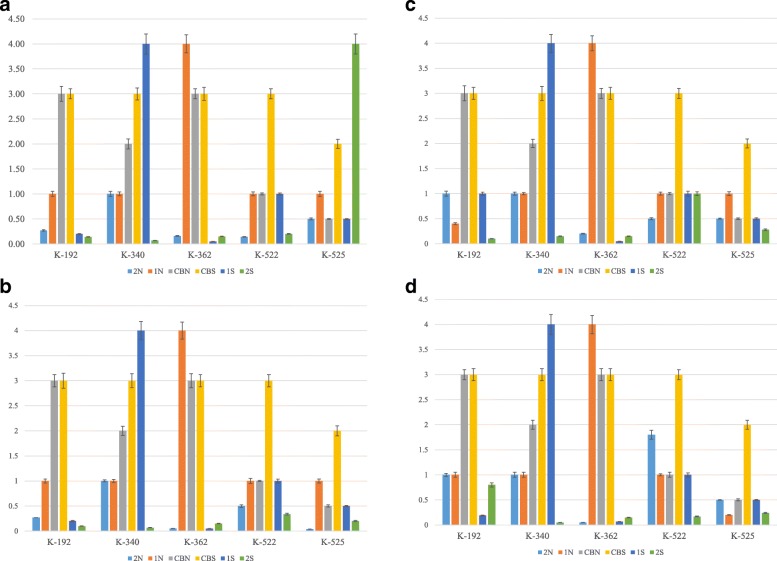
**a** – **d** The effect of preparations on the neuraminidase inhibition (NI50, mean mg/ml) of influenza virus strains H3N2 (**a**), H1N1 (**b**), H5N3 (**c**) and H7N1 (**d**). Tested viruses were diluted in enzyme buffer and aliquots of 25 μl of each dilution were added to the wells. As a positive control, enzyme buffer were used, instead of virus-containing material. After incubation for 30 min at 37 °C, 50 μl aliquot of substrate solution was added to each well and the plate was incubated at room temperature for 30 min. 150 μl aliquots of stop-solution buffer were added to the wells and optical density was measured by the “Tecan” spectrophotometer. The capacity of tested preparations to inhibit of neuraminidase activity of influenza viruses was estimated as effective concentration of tested preparations (EIC_50_, mg/ml) capable to inhibit 50% of neuraminidase activity (NI_50_)

**Fig. 3:**
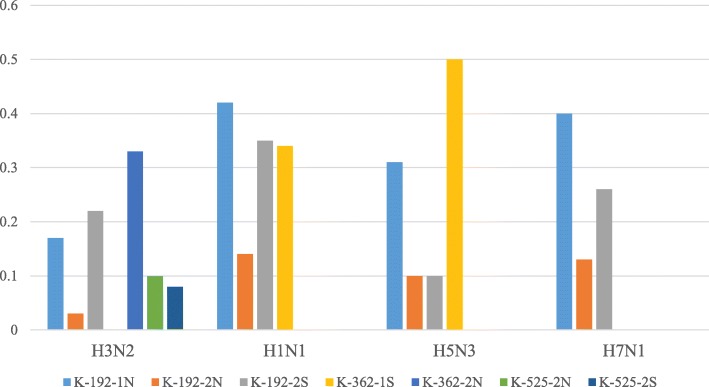
Effective Inhibition Concentration (EIC50, mg/ml) of preparations against influenza virus type A. Actinomycetes extracts, K-192-1 N, K-192-2 N, K-192-2S, K-362-1S, K-362-2 N, K-525-2 N, K-525-2S inhibited neuraminidase activity of H3N2, H1N1, H5N3 and H7N1. The actinomycetes extract K-362-1S inhibited the neuraminidase activity of influenza virus strains resistant to commercial antiviral drugs (A/Vladivostok/2/09, H1N1 and A/tern/South Africa/1/61, H5N3)

It was shown that 3 extracts of extremophilic actinomycetes: K-192-1 N, K-192-2 N and K-192-2S were capable to inhibit the neuraminidase activity of all tested strains of influenza virus A (Fig. 2a-d). Moreover, the extracts of actinomycetes K-362-2 N, K-525-2 N and K-525-2S inhibited the neuraminidase activity of epidemically significant strain of influenza virus, subtype H3N2 (Fig. 3).

The quantitative measurement of specific inhibition of neuraminidase activity in influenza virus H3N2 in 5 selected strains of actinomycetes is indicated by mean and standard deviation measures of neuraminidase inhibition (NI) of the H3N2 virus. As shown in Fig. 2a we observed that 5 selected strains grown in certain neutral or saline conditions were effective in inhibiting 50% of the neuraminidase activity of the influenza virus H3N2. K-192 and K-362 strains grown in 2 N, 1S and 2S conditions both required lower concentrations of preparations (and hence more active) to inhibit the neuraminidase activity of the H3N2 in comparison with 1 N, CBN and CBS conditions of growth (Fig. 2a). Among all 5 strains, the 2 N condition required relatively lowest amount of the extract to inhibit the neuraminidase activity, hence it is the most active preparation against H3N2 virus (Fig. 2a). On the other hand, the CBS as a condition is among the least effective in inhibiting neuraminidase activity for all 5 strains. Interestingly, the 2S preparation required small amounts of the extract in all strains except K-525, where it showed the highest concentration used, thus was the least active (Fig. 2a).

The smallest concentrations required to inactivate neuraminidase activity of the H1N1 were in the 6 preparations from strain K-525 (Fig. 2b). Preparations 2S and 2 N required fewer concentrations for all 5 strains to inhibit the neuraminidase activity in comparison with the remaining preparations (Fig. 2b). In 4 CBS preparations for all except for the K-525 strain, nearly the same concentrations were needed to inhibit neuraminidase activity of H1N1. Also, strains K-192 and K-362 showed relatively similar results for the effect on neuraminidase activity when grown in CBN (Fig. 2b). Overall, it can be observed that CBN and CBS preparations of the K-192, K-340 and K-362 exhibited the least activity for the extracts in inhibiting neuraminidase of H1N1 virus.

The highest (and hence the least effective) concentration in strains K-522 and K-525 required to inhibit neuraminidase activity of the H5N3 was from CBS preparation (Fig. 2c). In the remaining preparations, the CBS showed almost the same NI_50_ values, which were higher compared to 2 N and 2S conditions that had the lowest NI_50_ values from all 6 growth conditions in K-192, K-340, and K-362 (Fig. 2c). Overall, K-192-2S, K-340-2S, K-362-1S are the most active in inhibiting neuraminidase activity of the H5N3 since these extracts required the lowest concentrations (Fig. 2c).

The effect is close to the one observed for H5N3 and for H7N1 with slight differences in NI_50_ values for 1 N for K-525, 1S for K-192, 2 N for K-522 and 2S for K-522 (Fig. 2d). The most active extracts were for strains K-340 grown in 2S condition, K-362 in 2 N and 1Sconditions against H7N1 neuraminidase activity.

Figure 3 shows the EIC_50_ of tested preparations that were effective in inhibiting neuraminidase activity of the investigated influenza virus strains. Interestingly, one extract (K-362-1S) inhibited the activity of influenza virus strains resistant to commercial antiviral drugs (A/Vladivostok/2/09, H1N1 and A/tern/South Africa/1/61, H5N3).

**Fig. 4:**
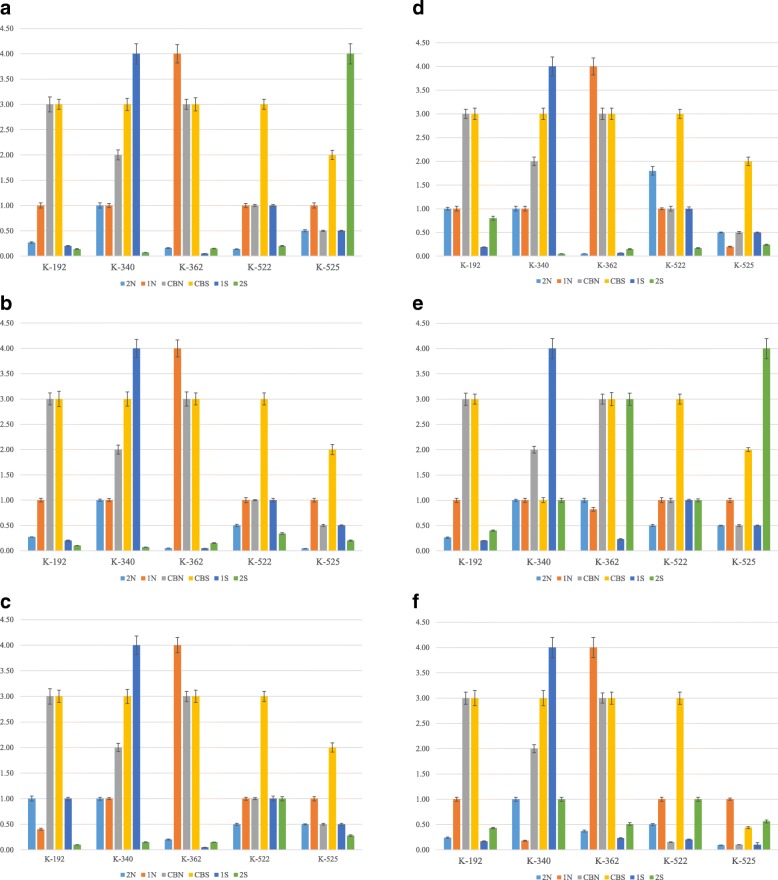
**a** - **f** Average effective virus-inhibiting concentration (EC_50_,mg/ml) of preprarations against influenza virus strains H3N2 (**a**), H1N1 (**b**), H5N3 (**c**), H7N1 (**d**) and PMV-1/Beadette (**e**), Sendai. strain 960 (**f**). Virus-inhibition activity of preparations obtained from extremophilic actinomycetes against Influenza virus H3N2 in dose 100 EID_50_/ml. The specific virus-inhibitory activity of the studied preparations was determined in accordance with the guidelines of the “Pre-clinical Drug Research Guide” [21, 22]. Various doses of extract preparations were mixed with an equal volume of 100 EID50/ml of the tested virus. After 30 min of incubation at 370 С, the mixture was inoculated into the chorionic-allantoic cavity of 10-days old chicken embryos. Viruses were grown for 24–72 h at 370С depending on the viral strain. The presence of the viruses was indicated by the hemagglutination test (HA-test). The ability of investigated preparations to inhibit virus infectious activity was estimated by comparison of the results of HA-test in experimental and control samples. As a control, the buffered saline pH 7.2, was used and the allantoic fluid with viruses but without the addition of preparations obtained from extremophilic actinomycetes

The middle effective concentration of tested preparations (EC_50_, mg/ml) that caused 50% inhibition of virus infectious activity was determined (Fig. 4a-f). Extracts of CBN and CBS preparations demonstrated lower virus-inhibition activity against all investigated strains of influenza virus and paramyxoviruses in comparison with the extracts of biomass of extremophilic actinomycetes such as K-362-2 N, K-192-2S, K-340-2S and K-362-2S (Fig. 4a-f). Overall, the biomass preparations of most (but not all) strains grown in saline (2S) conditions were found to be most effective in inhibiting the Influenza A viral subtype, PMV-1/Beadette and Sendai. strain 960, followed by the 1S preparations (extracts of culture broth of strains grown in saline (extreme) conditions). Results from these Figures were further analyzed for more meaningful interpretation to determine the SI-index as described in the section below.

### SI-index of actinomycetes preparations to determine the effective viral inhibiting activity

Further in experiments on chicken embryos, the effective dose of preparations capable of inhibiting 50% of reproduction of tested viruses was estimated. The selective index (SI-index) against ortho- and paramyxoviruses, determined by the ratio of the average toxic substance concentration (TC_50_) to the average effective virus-inhibiting concentration (EC_50_), was calculated accordingly the guidelines (“Pre-clinical Drug Research Guide”). The results of the analysis are presented for preparations that possessed marked antiviral properties, showing SI-index greater than or equal to 10 (Table 2).

It was established that the culture broth of actinomycetes strains K-192, K-340 and K-362 (having SI index of 0) were not able to inhibit the reproduction of all investigated influenza virus strains and paramyxoviruses. Four extracts of culture broth of actinomycetes strain K-192-1 N, K-340-2 N, K-340-1S and K-522-1 N also did not express antiviral activity in the tested interval of doses (having SI index of 0). However, culture broth of actinomycetes strains K-525 and K-522, and actinomycetes extract K-522-1S, K-525-1S, K-525-1 N, K-522-2 N, K-340-1 N had good SI-index in the relation of at least one of tested viruses (Table 2).

Thus, extract of the biomass of extremophilic actinomycetes strain K-362, grown in neutral conditions of cultivation (K-362-2 N) demonstrated high virus-inhibition activity against all investigated strains of influenza virus (Table 2). Also, it was found that 3 extracts of the biomass of extremophilic actinomycetes, strains K-192, K-340 and K-362 grown in the presence of high salt concentration (extracts K-192-2S, K-340-2S, K-362-2S) possessed high antiviral activity against all investigated strains of influenza virus, including the drug-resistant strains (Table 2).

An SI value that is higher than 10 is considered a good selective index that can give an idea about the selectivity of actinomycetes preparations on selectively killing the virus, and not the host. Overall, specific antiviral action of K-340-2S, K-362-2 N, K-362-1S, and K-362-2S had shown SI-index to be more than 10 against all strains of influenza type A viruses investigated. Additionally, both K-362-1S and K-362-2S had the highest SI with a value of 40 against H3N2, H1N1, and H5N3. Only K-192-1S and K-192-2S extracts had good antiviral specificity result against PMV-1/Beadette, SI being 10, whereas the effective SI against Sendai, strain 960 was found in K-192-1S, K-340-1 N, K-362-2S, K-522-CBN, K-522-1S, K-525-CBN, K-525-2 N, K-525-1S and K-525-2S.

### Comparison of actinomycetes preparations with commercial antiviral drugs and plant preparations

**Fig. 5:**
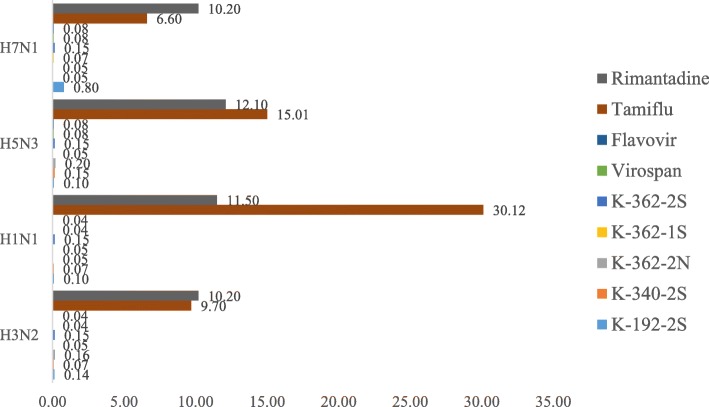
Effective Inhibition Concentration (EIC50, mg/ml) of preparations against H7N1, H5N3, H1N1, H3N2 in comparison with 2 commercially available antiviral drugs, Rimantadine and Tamiflu. Also, the inhibition of viral activity by studied extracts was comparable with plant antiviral preparations Virospan and Flavovir

**Fig. 6:**
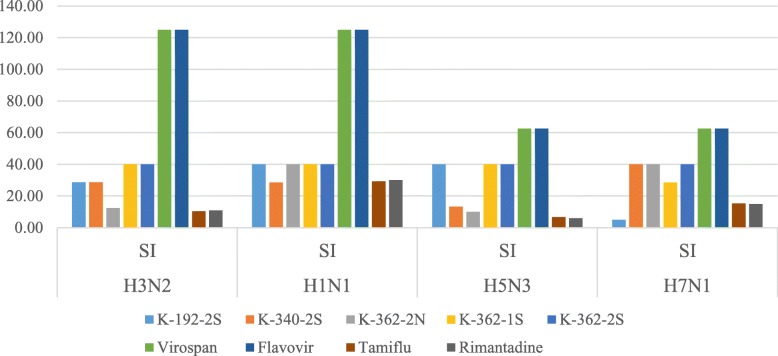
Specific antiviral action (SI) of tested preparations on influenza viruses. Commercially available drugs Virospan and Flavovir showed the highest SI values against all influenza viruses studied. However, extracts K-192-2S, K-340-2S, K-362-2 N, K-362-1S, had higher SI than Tamiflu and Rimantadine against H3N2, H1N1, H5N3, and H7N1 viral strains

We found that the virus-inhibition activity of extremophilic actinomycetes extracts K-192-2S, K-340-2S, K-362-2S, K-362-2 N, and K-362-1S is comparable with antiviral activity of commercial anti-flu drugs Tamiflu and Rimantadine and plant antiviral preparations Virospan and Flavovir (Figs. 5 and 6).

Actinomycetes preparations required lower concentrations to effectively kill 50% of viruses in comparison with the two commercially available antiviral drugs, Rimantadine and Tamiflu.

SI values were the highest for the plant origin preparations Virospan and Flavovir against all model influenza A viruses studied. However, the preparations of actinomycetes, specifically K-192-2S, K-340-2S, K-362-2 N, and K-362-1S, had higher SI than commercially available drugs Tamiflu and Rimantadine against H3N2, H1N1, H5N3, and H7N1 influenza A viruses.

## Discussion

We found in this study that several extracts from strains K-192, K-340 and K-362 (preparations K-192-2S, K-340-2S, K-362-2 N, and K-362-1S) exhibited significant antiviral activities against the tested influenza viruses displaying antiviral SI values higher than those obtained by testing with the commercially available drugs Tamiflu and Rimantidine. In general, samples of the actinomycetes coming from CBN and CBS had lower toxicity than other preparations against studied ortho- and paramyxoviruses. Comparison of virus-inhibition activity of selected extracts of extremophilic actinomycetes with virus-inhibition properties of commercial anti-flu drugs Rimantadine and Tamiflu and herbal preparations “Virospan” and “Flavovir”, have shown that all selected extracts of extremophilic actinomycetes were comparable in anti-viral activity with commercial and plant preparations by the indices of selective index (Table 2).

It was assumed that with all variables and observations being independent, the results obtained showed true effect because the methodology used to conduct all experiments was based on standard protocols and guidelines, and thus random errors were minimized. We found that the toxicity of investigated actinomycetes preparations depended on the conditions of cultivation and varied on different biological models used for toxicity hemolytic activity testing. The lower toxicity was observed on the model of chicken embryos, whereas the higher one was on the culture of chicken fibroblasts cells during hemolytic activity testing. Overall, samples of actinomycetes culture broth possessed the lower toxicity in comparison with the extracts of actinomycetes biomass. The results of toxicity testing which were found not to be significantly different based on the output of the Kruskal-Wallis H test (comparing the 3 assays used) were used to determine the intervals of doses suitable for antiviral activity analysis. This was based on the SI values (calculated based on the ratio of average toxic substance concentration (TC_50_) to average effective virus-inhibiting concentration (EC_50_)). Similar to the approach we described here for our study, many other researchers have modified nutrients and other physicochemical factors, and also used statistical optimization approaches to enhance production and identification of several bioactive compounds [11]. Interestingly, a set of 13 antiherpetic antiviral derivatives known as fattiviricins identified from the producer organism *Streptomyces microflavus,* were also shown to have potent activity against some enveloped RNA viruses such as the influenza A and B viruses [14]. AH-135Y produced from the culture filtrate of *Streptomyces albovinaceus* strain number AH-135 which was reported to be a new glutarimide antibiotic, was shown to be active against HSV-1 as well as influenza virus A and RSV [14]. However thus far no study has reported antiviral activities against our tested viral strains in extracts from actinomycetes strains grown in-vitro in saline and alkaline culture conditions, enhancing the possibility of the novelty of active compounds within our active extracts. Furthermore, our preliminary investigations on the identity of producer strains with antiviral activities that we have in our collection (data not presented here) do not include the two strains reported here (*Streptomyces microflavus* and *Streptomyces albovinaceus*), further supporting the idea that our active antiviral compounds described in this study are novel.

Hemagglutination activity testing showed inhibition of only one influenza virus strain- H1N1. The least concentration of 2S preparations in K-192, K-340 and K-522 and 2 N preparations in K-362 and K-525 was required to inhibit hemagglutination activity, thus indicating high effectiveness. However, the CBS preparations of the most strains showed less effectiveness in inhibiting hemagglutination activity of H1N1. The K-192-1 N, K-192-2 N, and K-192-2S extracts were able to suppress the neuraminidase activity of all tested influenza virus strains. In addition, extracts K-192, K-362 in 2 N, 1S and 2S conditions had the ability to suppress the neuraminidase activity of epidemic-significant H3N2 influenza virus strain. Also, these strains were more effective in neuraminidase inhibition than in 1 N, CBN and CBS conditions. Overall, 2S and 2 N preparations were the most effective in neuraminidase inhibition of influenza virus H1N1 for all strains, while the most effective strain was K-525. Moreover, K-362-1S extract suppressed neuraminidase activity of influenza viruses A/Vladivostok/2/09 (H1N1) and A/tern/South Africa/1/61 (H5N3) resistant to commercial antiviral drugs. Interestingly one recent study reported the identification of an antiviral butanolide from the culture broth of *Streptomyces* sp. SMU03 which inhabit the intestines of *Elephas maximus*, which displayed broad and potent activity against a panel of influenza viruses including the H1N1 and H3N2 subtypes [15]. Given that this compound was found to interfere with the early stage of influenza A virus lifecycle, it was postulated that the target could be the HA2 subunit of hemagglutinin enzyme. From the results of our study, we found that most of our preparations that exhibited antiviral activities were effective to suppress the neuraminidase activity, and not the hemagglutinin enzyme activity.

Analysis of the virus-inhibition has shown that 8 preparations: culture broth of strains K-525 and K-522 and actinomycetes extracts K-522-1S, K-525 -1S, K-525-1 N, K-522-2 N, K-362-1 N, and K-340-1 N possessed significant virus-inhibition activity against ortho- and paramyxoviruses. The highest antiviral activity was demonstrated within 5 extracts, which were K-192-2S, K-340-2S, K-362-2 N, K-362-2S, and K-362-1S. These extracts can block the reproduction of all investigated strains of influenza virus. Overall, 2S extracts of actinomycetes demonstrated a higher antiviral activity than CBS extracts of all actinomycetes strains except K-525, when investigated on influenza type A and paramyxoviruses. However, the K-525 strain was the most effective in inhibiting Sendai strain 960 for all 6 extracts, when compared with other actinomycetes strains. Fifteen preparations obtained from actinomycetes, cultivated under extreme and neutral conditions, had SI-index greater than or equal to 10, thus indicating good antiviral specificity. Even when the exact composition of the active components within the extracts are yet to be identified, we have demonstrated in this preliminary study the great potential of discovering novel antiviral compounds that can be further characterized to produce therapeutically effective antivirals for treatment of infections caused by our tested viral strains. As previously mentioned, several studies reported antiviral activities of natural compounds that originated from Actinobacteria whereby some of the antiviral mechanisms of actions of these compounds have been elucidated [11–14]. A recent study reported that one of the two novel antimicrobial compounds isolated from the culture broth of *Streptomyces* sp. AM-2504 showed antiviral activity against dengue virus [16]. As summarized in one report, Antimycin A1a isolated from *Streptomyces kaviengensis* was found to disrupt mitochondrial transport and pyrimidine biosynthesis in WEEV and that Ahmpatinin ‘Bu from *Streptomyces* sp. CPCC 202950 inhibit the activity of HIV protease [11]. Yet another study showed that high-mannose type saccharide chains of gp120 are the molecular targets of actinohivin, which was isolated from *Longispora albida* which is a relatively new actinomycetes species, in its anti-HIV activity [12].

Even with the significant findings summarized here, there are some limitations of this study which we are currently addressing and incorporating for designs of future studies. The active compounds responsible for these antiviral activities are yet to be identified but we are currently working towards the goal of identification of the producer strains, active antiviral compounds within the extracts and elucidation of the antiviral mechanism of actions. Since this is a preliminary screening study of selected extracts from our collection, it represents the first step in our efforts to identify extracts and producer strains with promising potential to be further researched for development of efficacious antiviral therapeutic agents. The strength and uniqueness here is that this represents the only study reporting antiviral activity from actinomycetes producer strains that are also extremophiles grown in optimized culture environment that mimics the environment that these strains were isolated from. K-192-2S, K-340-2S, K-362-2 N, K-362-2S and K-362-1S extracts were comparable with virus-inhibition properties of herbal preparations “Virospan” and “Flavovir”.

Our previous study showed that the plant extracts Virospan and Flavovir were prepared by extraction of plant tissues using 5 ethyl acetate and ethyl alcohol [26, 27]. The final preparations tested in in-vitro experiments on chicken embryos showed that Virospan in a dose of 0.2 mg inhibited reproduction of influenza A virus, specifically H5N3 and H3N2 subtypes by 40%, and by 60% when 1.0 mg of the preparation was added [27]. Additionally, in-vivo experiments showed that residual infectivity of influenza A virus H3N2 in the lungs of infected mice after addition of 200 micrograms of the Flavovir extract decreased by 0.501 g. These results were comparable with the effect of Relenza and Remantadin, while Tamiflu showed a decrease in 0.71 g [27]. The fact that our extracts with comparable activities to our established plant extracts Virospan and Flavovir which also showed very good SI index is highly promising for further identification and characterization. Therefore our extracts should be developed further potentially as an effective antiviral medication. A similar study reported active antiviral activity of 27 plant extracts from Nigeria against 3 serotypes of echoviruses which suggested the significance of these extracts to be further developed as therapeutic antiviral agents [28].

The most promising results were that, extract preparations K-192-2S, K-340-2S, K-362-2 N, and K-362-1S in this study exhibited higher SI values compared to Rimantadine and Tamiflu, which are well established antivirals with the mechanism of antiviral actions on viral replicative cycle and inhibiting the neuraminidase enzyme, respectively [29, 30]. Our findings thus indicate that these extracts are great potential producers of new antiviral drugs. One of the possible mechanisms of antiviral action would be as inhibitors of the neuraminidase enzyme found on the influenza virus surface which would prevent budding from the host cell, viral replication, and infectivity.

## Conclusions

Overall, we found that the strains of extremophilic actinomycetes K-192, K-340, and K-362 are interesting for further research as possible producers of biologically active substances with antiviral activity. Based on results of SI indexes higher than 10, it can be suggested that this study identified specific preparations of extremophilic actinomycetes that can be considered as a promising source for isolation and study of compounds that can block the reproduction of influenza virus type A. Future studies will focus on isolation and characterization of active compounds responsible for the antiviral actions followed by elucidation of the mechanism of action for the viral inhibitory activities.

## Supplementary information

**Additional file 1: Figure S1.** The dose of actinomycetes preparations inducing 50% lysis of erythrocytes (ТC_50_ mean value, mg/ml).

**Additional file 2: Figure S2.** The dose of actinomycetes preparations inducing 50% death of chicken fibroblasts (ТC_50_ mean value, mg/ml).

**Additional file 3: Figure S3.** The dose of actinomycetes preparations inducing 50% death of chicken embryos (ТC_50_ mean value, mg/ml).

**Additional file 4: Table S1.** Kruskal-Wallis equality-of-populations rank test on the dose of actinomycetes preparations inducing 50% lysis/death of erythrocytes/chicken fibroblasts /chicken embryos (ТC_50_ mean value, mg/ml).

## Data Availability

All data generated or analysed during this study are included in the manuscript, its supplementary information files and available from the corresponding author on reasonable request.

## Abbreviations

1 N: extracts prepared from culture broth of actinomycetes strains grown in neutral growth conditions
1S: extracts prepared from culture broth of actinomycetes strains grown in saline growth conditions
2 N: extracts prepared from actinomycetes strain biomass (cells) in neutral conditions of growth
2S: extracts prepared from actinomycetes strain biomass (cells) in saline conditions of growth
CBN: Culture Broth Neutral
CBS: Culture Broth Saline
EIC_50_: Effective inhibition concentration of preparations
EC_50_: Average/middle effective virus-inhibiting concentration
HI_50_: concentration of substance that induced 50% of hemagglutination inhibition
NI_50_: concentration of substance that induced 50% of neuraminidase inhibition
ТC_50_: Toxic substance concentration that induced 50% cell lysis
