# Supplementary figures and images for: Antiviral activities of extremophilic actinomycetes extracts from Kazakhstan’s unique ecosystems against influenza viruses and paramyxoviruses
Source: Virol J. 2019 Dec 2;16:150. doi: 10.1186/s12985-019-1254-1 (PMC6889349; doi:10.1186/s12985-019-1254-1)

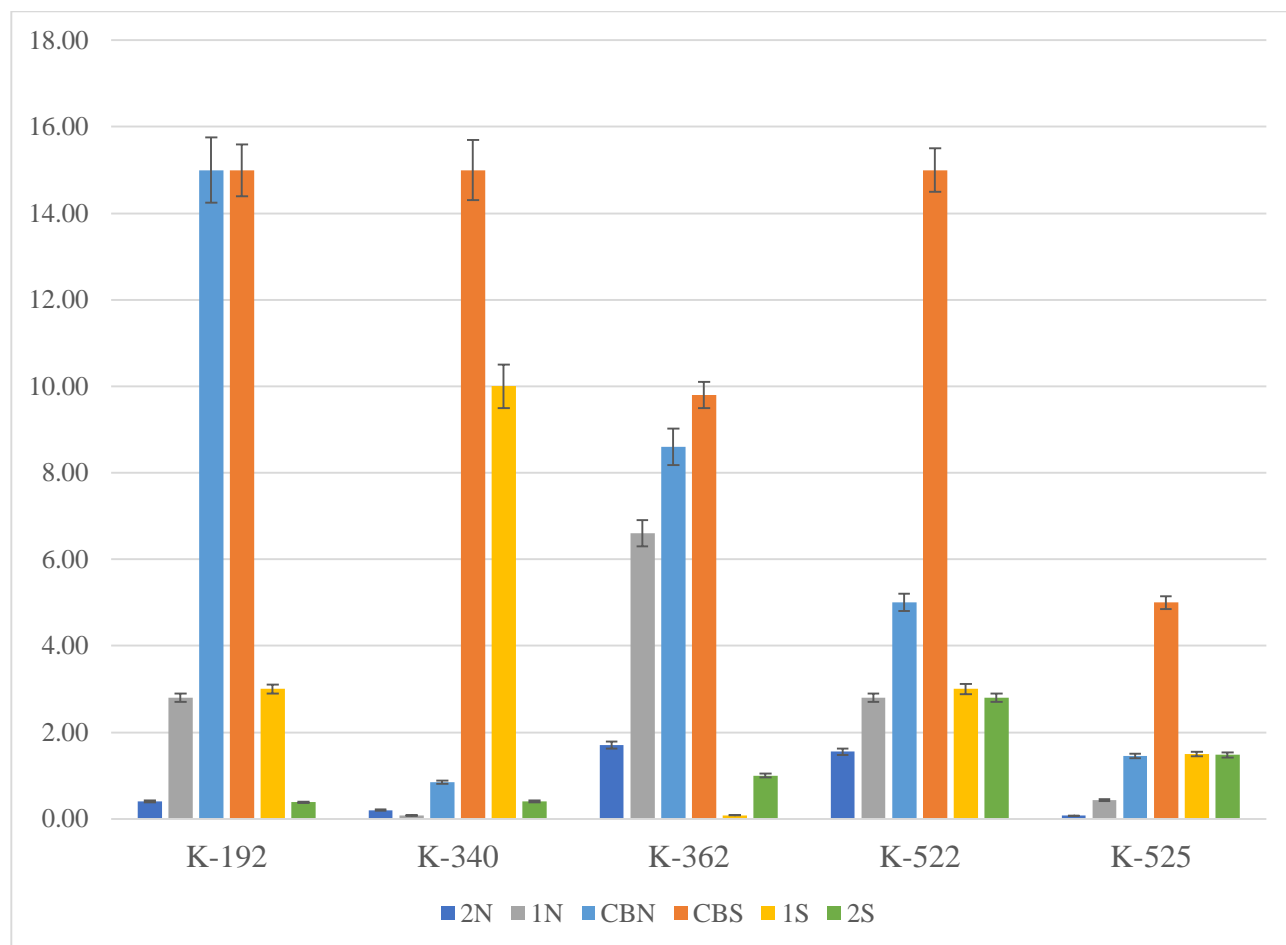

Supplement: Supplementary file 1 — Additional file 1: Figure S1. The dose of actinomycetes preparations inducing 50% lysis of erythrocytes (ТC50 mean value, mg/ml). [file 12985_2019_1254_MOESM1_ESM.pdf]

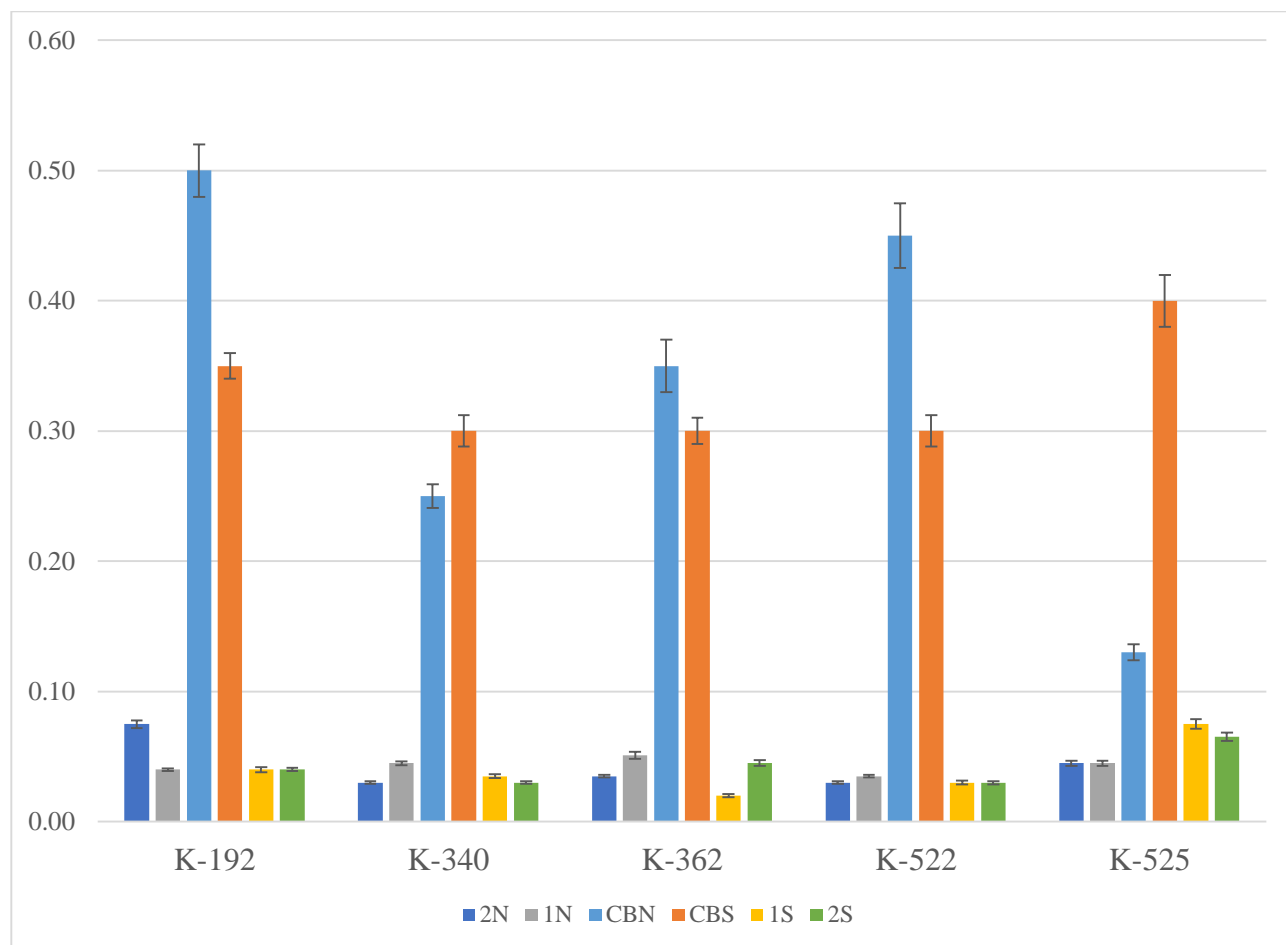

Supplement: Supplementary file 2 — Additional file 2: Figure S2. The dose of actinomycetes preparations inducing 50% death of chicken fibroblasts (ТC50 mean value, mg/ml). [file 12985_2019_1254_MOESM2_ESM.pdf]

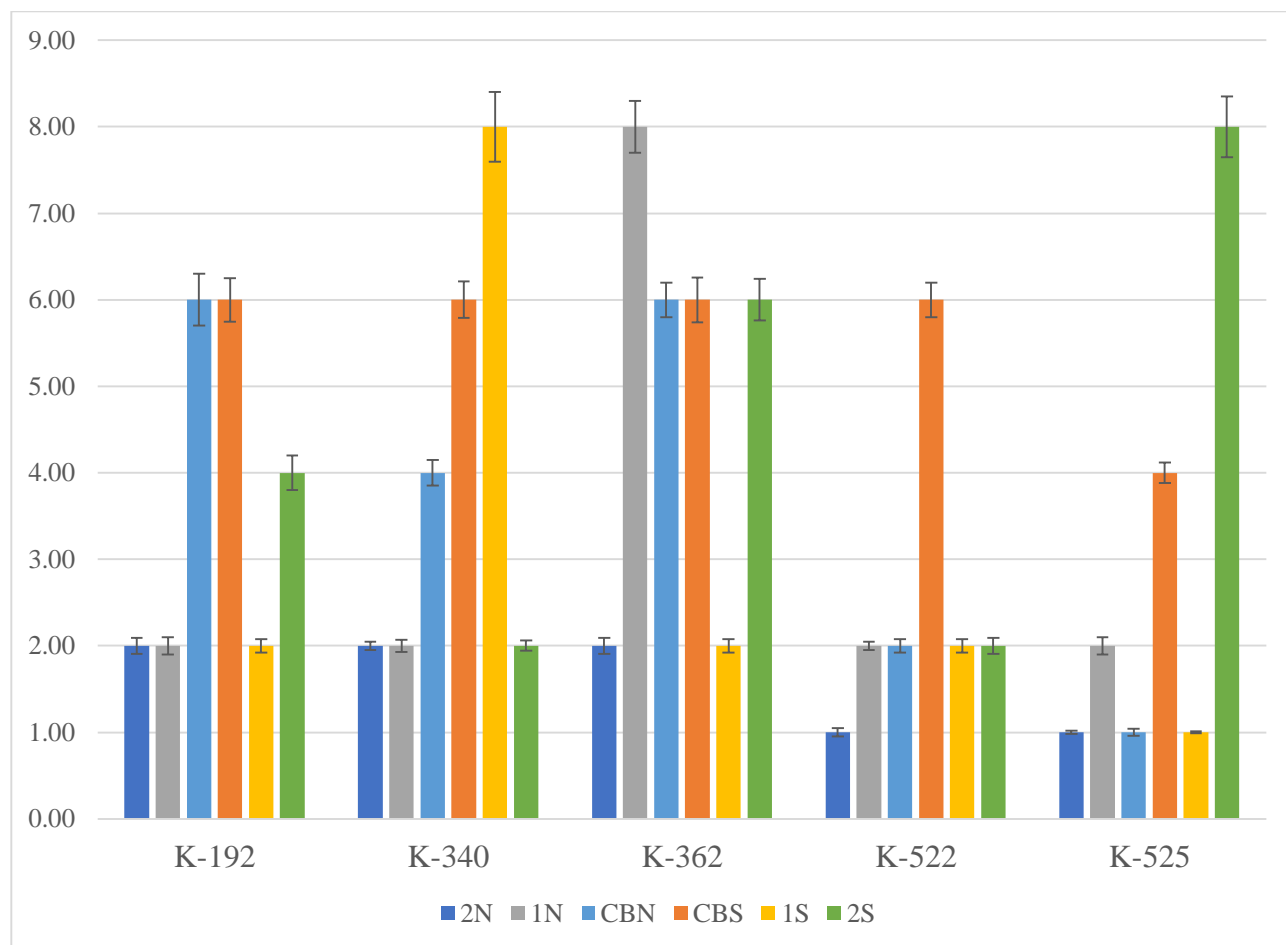

Supplement: Supplementary file 3 — Additional file 3: Figure S3. The dose of actinomycetes preparations inducing 50% death of chicken embryos (ТC50 mean value, mg/ml). [file 12985_2019_1254_MOESM3_ESM.pdf]
